# Hyperglycemia-Induced Protein Kinase C Activation Inhibits Phagocytosis of C3b- and Immunoglobulin G–Opsonized Yeast Particles in Normal Human Neutrophils

**DOI:** 10.1155/EDR.2003.125

**Published:** 2003

**Authors:** Daniel Saiepour, Janove Sehlin, Per-Arne Oldenborg

**Affiliations:** 1 Department of Integrative Medical Biology Section for Histology and Cell Biology Umeå University Umeå SE-901 87 Sweden

## Abstract

The aim of this study was to investigate the effects of elevated glucose concentrations on complement receptor– and Fc*γ* receptor–mediated phagocytosis in normal human neutrophils. D-Glucose at 15 or 25 mM dose-dependently inhibited both complement receptor– and Fc*γ* receptor– mediated phagocytosis, as compared to that at a normal physiological glucose concentration. The protein kinase C (PKC) inhibitors GF109203X and Go6976 both dose dependently and completely reversed the inhibitory effect of 25 mM D-glucose on phagocytosis. Complement receptor– mediated phagocytosis was dose-dependently inhibited by the cell permeable diacylglycerol analogue 1,2-dioctanoyl*sn*- glycerol (DAG), an effect that was abolished by PKC inhibitors. Furthermore, suboptimal inhibitory concentrations of DAG and glucose showed an additive inhibitory effect on complement receptor–mediated phagocytosis. The authors conclude that elevated glucose concentrations can inhibit complement receptor and Fc*γ* receptor–mediated phagocytosis in normal human neutrophils by activating PKC*α* and/or PKC*β*, an effect possibly mediated by DAG.


*Experimental Diab. Res.*, 4:125–132, 2003
Copyright © Taylor and Francis Inc.
ISSN: 1543-8600 print / 1543-8619 online
DOI: 10.1080/15438600390228487

### Hyperglycemia-Induced Protein Kinase C Activation Inhibits Phagocytosis of C3b- and Immunoglobulin G–Opsonized Yeast Particles in Normal Human Neutrophils

**Daniel Saiepour, Janove Sehlin, and Per-Arne Oldenborg**

*Department of Integrative Medical Biology, Section for Histology and Cell Biology, Umeå University, Umeå, Sweden*

**The aim of this study was to investigate the effects of elevated glucose concentrations on complement receptor– and Fc$\gamma$ receptor–mediated phagocytosis in normal human neutrophils. D-Glucose at 15 or 25 mM dose-dependently inhibited both complement receptor– and Fc$\gamma$ receptor–mediated phagocytosis, as compared to that at a normal physiological glucose concentration. The protein kinase C (PKC) inhibitors GF109203X and Go6976 both dose-dependently and completely reversed the inhibitory effect of 25 mM D-glucose on phagocytosis. Complement receptor–mediated phagocytosis was dose-dependently inhibited by the cell permeable diacylglycerol analogue 1,2-dioctanoyl-*sn*-glycerol (DAG), an effect that was abolished by PKC inhibitors. Furthermore, suboptimal inhibitory concentrations of DAG and glucose showed an additive inhibitory effect on complement receptor–mediated phagocytosis. The authors conclude that elevated glucose concentrations can inhibit complement receptor and Fc$\gamma$ receptor–mediated phagocytosis in normal human neutrophils by activating PKC$\alpha$ and/or PKC$\beta$, an effect possibly mediated by DAG.**

**Keywords** Diabetes; Diacylglycerol; Glucose; PKC; Polymorphonuclear leukocytes

Received 11 February 2003; accepted 8 May 2003.

This work was supported in part by the Swedish Medical Research Council (12x-4756, 06P-14098), the Swedish Association for Medical Research, the Swedish Diabetes Association, the Sahlberg Foundation, and the Swedish Society of Medicine.

Address correspondence to Dr. Per-Arne Oldenborg, Department of Integrative Medical Biology, Section for Histology and Cell Biology, Umeå University, SE-901 87 Umeå, Sweden. E-mail: per-arne.oldenborg@histocel.umu.se

An important complication in diabetes mellitus is an increased sensitivity to infection, leading to many secondary diseases [1]. This may in part be explained by an impaired function of neutrophil granulocytes [2, 3]. In fact, numerous studies of neutrophils from patients with diabetes mellitus have reported defects in many important functional steps, such as adherence, chemotaxis, respiratory burst, phagocytosis, and bacterial killing [4–7]. Although defective neutrophil function seems to be closely related to poorly controlled diabetes and high glucose levels, the cellular/molecular mechanisms underlying the impaired neutrophil function in diabetes are still not well understood.

Effective phagocytosis of invading pathogens by neutrophils is of significant importance for successful resistance to infectious diseases [8]. Phagocytosis is a complicated process that can be activated by several different types of prophagocytic receptors, where two of the best characterized are Fc$\gamma$ receptors for the Fc portion of immunoglobulin G (IgG) on IgG-opsonized targets and complement receptor 3 (CR3) that will recognize C3bi-opsonized targets [8]. Conflicting data have been reported about the in vitro phagocytic capacity of neutrophils from patients with diabetes mellitus, showing both normal [5–7] as well as decreased phagocytosis activity [2, 9–11]. Interestingly, increased glucose concentrations were found to impair phagocytosis of IgG-opsonized *Staphylococcus aureus* by normal human neutrophils [12], whereas phagocytosis of C3bi-opsonized *Staphylococcus aureus* was unaffected by elevated glucose concentrations [13]. These findings suggest that Fc$\gamma$ receptor–mediated phagocytosis is more sensitive to elevated glucose concentrations than is CR3-mediated phagocytosis. However the exact mechanisms by which hyperglycemia may affect the phagocytic capacity of neutrophils is still unclear.

The aim of this study was to investigate the effects of elevated glucose concentrations on phagocytosis of C3bi- and IgG-opsonized yeast particles in normal human neutrophils.

### MATERIALS AND METHODS

### Chemicals

Electrophoretically homogenous, lyophilized bovine serum albumin (BSA) was from Boehringer Mannheim Scandinavia AB, Stockholm, Sweden. GF109203X was from Calbiochem, La Jolla, CA, USA. Go6976 was from LC Laboratories, Woburn, MA, USA. May-Grünwalds reagent was from Apoteksbolaget, Malmö, Sweden. 1,2-Dioctanoyl-*sn*-glycerol (DAG), fluorescein isothiocyanate (FITC) mixed polymers, and phorbol 12-myristate 13-acetate (PMA) were from Sigma Chemical, St. Louis, MO, USA. Polymorphprep was from Nycomed Pharma, Oslo, Norway. Rabbit anti-yeast IgG was a generous gift of Dr. Maria Fällman, Umeå University, Sweden. Crystalline tannic acid was from Ketsen, Stockholm, Sweden. PMA, GF109203x and Go6976 were dissolved in dimethylsulfoxide (DMSO) and stored at −20°C. The highest final DMSO concentration in the experiments was 0.1% (*v*/*v*) and equal concentrations of DMSO were included in the controls. Double-distilled water was used throughout and all reagents used were of analytical grade.

### Media

Krebs-Ringer Hepes (KRH) medium containing (in mM): NaCl 136, KCl 4.7, $KH_2PO_4$ 1.2, $MgSO_4$ 1.2, $NaHCO_3$ 5, Hepes 20, D-glucose 5, $CaCl_2$ 1.1, supplemented with 1 mg/mL BSA, pH 7.40, was used in all experiments. Phosphate-buffered saline (PBS) containing (in mM): $Na_2HPO_4$ 8.1, KCl 2.7, $KH_2PO_4$ 1.5, and NaCl 137, pH 7.40, was used to wash the cells during isolation and staining.

### Isolation of Neutrophil Granulocytes

Heparinized venous blood was obtained from healthy adult volunteers attending the Blood Bank at the Umeå University Hospital. The study was performed in accordance with the ethical standards of the 1964 Declaration of Helsinki and all blood donors gave their informed verbal consent. Neutrophil granulocytes were isolated essentially using the method of Böyum [14]. In short, blood was layered on top of Polymorphprep and spun in a centrifuge at 450 to 500 × *g* and room temperature for 30 minutes. The neutrophil layer was then collected and following a brief hypotonic lysis of remaining erythrocytes, using distilled water, the neutrophils were washed 3 times in PBS at 200 × *g* for 5 minutes and finally suspended at $1 \times 10^6$ cells/mL in KRH. The cell suspension was kept at room temperature until used, which was within 1 hour.

### Preparation and Opsonization of Yeast Particles

Opsonized yeast particles were prepared essentially as previously described [15]. For complement opsonization, heat killed FITC-labeled or unlabeled yeast particles were incubated with 20% normal human serum in KRH at 37°C for 30 minutes. For IgG opsonization, heat killed yeast particles were incubated with purified rabbit anti-yeast IgG (24 $\mu$g/mL) in the presence of 20% heat-inactivated (56°C, 30 minutes) human serum in KRH at 37°C for 30 minutes. All particles were then washed twice, resuspended at $1 \times 10^8$/mL in KRH, and kept on ice until used, which was within 2 hours.

### Measurement of Phagocytosis

For measurements of phagocytosis, neutrophils ($1 \times 10^6$/mL) were first incubated in suspension with 5, 15, or 25 mM D-glucose at 37°C for 30 minutes. DAG, protein kinase C (PKC) inhibitors, and PMA, when used, were added during the last 5 minutes prior to the addition of yeast particles. C3b-opsonized yeast (C3bY) or IgG-opsonized yeast (IgGY) particles were then added to the neutrophils, which were further incubated at 37°C for 15 minutes. The tubes were then put on ice for 15 minutes to stop the phagocytosis process. Phagocytosis was evaluated either by light microscopy according to the method by Giaimis and colleagues [16] or by flow cytometry using FITC-labeled yeast particles [15]. For light microscopic evaluation of phagocytosis, neutrophils from each sample were attached to glass slides. To visually separate ingested yeast particles from extracellularly bound, noningested particles [16], slides were then treated with 1% (*w*/*v*) tannic acid in PBS for 1 minute at room temperature. Next, slides were stained with May-Grünwalds reagent for 5 minutes and finally washed with $H_2O$ for another 5 minutes. Tannic acid will not get access to ingested yeast particles, but will only etch the extracellularly bound yeast. Therefore, by using this method, ingested yeast particles will have a light pink color whereas extracellularly bound yeast will have a dark purple color. The slides were allowed to dry at room temperature before microscopic evaluation. The number of ingested and extracellularly bound particles was counted by light microscopy. One hundred neutrophils were analyzed in each of 3 separate preparations from each sample/incubation condition. Phagocytosis was expressed as a phagocytosis index showing number of ingested yeast particles per 100 neutrophils.

For flow cytometric evaluation of phagocytosis, opsonized FITC-labeled yeast particles were used in the phagocytosis assay, as described above. Following phagocytosis, samples on ice were mixed with trypan blue (1% final concentration) to quench the fluorescence of yeast particles that were not fully ingested by the neutrophils. Phagocytosis was quantified by using FACscan flow cytometry (BD Biosciences, Mountain View, CA, USA) and analyzed using CellQuest software (BD Biosciences). Sufficient quenching of noningested yeast was confirmed by comparing the fluorescence signal from FITC yeast, with or without trypan blue. The results obtained by the 2 different methods described above gave virtually identical results.

### Statistics

Statistical analyses were performed by using the two-tailed Student's *t* test for paired or unpaired samples (see legends to the figures and table). All results are expressed as mean ± SEM.

### RESULTS

### Elevated Glucose Concentrations Inhibit Ingestion of C3b- and IgG-Opsonized Yeast Particles

To study the effects of elevated glucose concentrations on complement receptor-mediated phagocytosis, C3bY were presented to neutrophils in suspension. Following a 30-minute preincubation with 15 mM or 25 mM glucose, phagocytosis of C3bY was dose-dependently reduced to 73.2% ± 4.7% and 42.5% ± 2.7% of that seen at 5 mM glucose ($P < .01$ and $P < .001$ respectively), (Figure 1*A*). We next studied the effects of elevated glucose concentrations on Fc$\gamma$ receptor–mediated phagocytosis, by using IgGY. Also here, phagocytosis was dose-dependently reduced by 15 and 25 mM glucose to 66.8% ± 6.8% and 52.6% ± 8.1% of that at 5 mM glucose ($P < .01$ and $P < .001$ respectively) (Figure 1*A*). When the role of the C3bY/neutrophil ratio was studied, we found a slight reduction of the inhibitory effect of 15 or 25 mM glucose when the C3bY/neutrophil ratio was increased (Figure 2*A*, *B*). Thus, elevated glucose concentrations can dose-dependently inhibit phagocytosis in normal human neutrophils.

The inhibitory effect of glucose on neutrophil phagocytosis could be the result of either a reduced binding of C3bY or IgGY to neutrophil complement or Fc$\gamma$ receptors, or due to a reduced ingestion of yeast particles bound to the cell surface, or both. The total number of neutrophil-associated yeast particles (C3bY), both ingested and bound to the outside of the neutrophils, was affected by glucose to a rather low extent (232 ± 8 C3bY/100 neutrophils at 25 mM glucose versus 265 ± 10 C3bY/100 neutrophils at 5 mM glucose; $P < .05$) (Table 1). When the total number of cell-associated IgGY was analyzed in the same way, there was a nonsignificant decrease in the total number of cell-associated IgGY in the presence of 25 mM glucose (144 ± 37 IgGY/100 neutrophils), as compared to that at 5 mM glucose (194 ± 12 IgGY/100 neutrophils) ($P > .05$; Table 1).

The small effects of elevated glucose concentrations on the total number of neutrophil-associated yeast particles could be explained by the glucose-induced reduction in phagocytosed particles and not by an impairment of the binding of yeast particles to complement or Fc$\gamma$ receptors. It is therefore most likely that the inhibitory effects of elevated glucose concentrations are

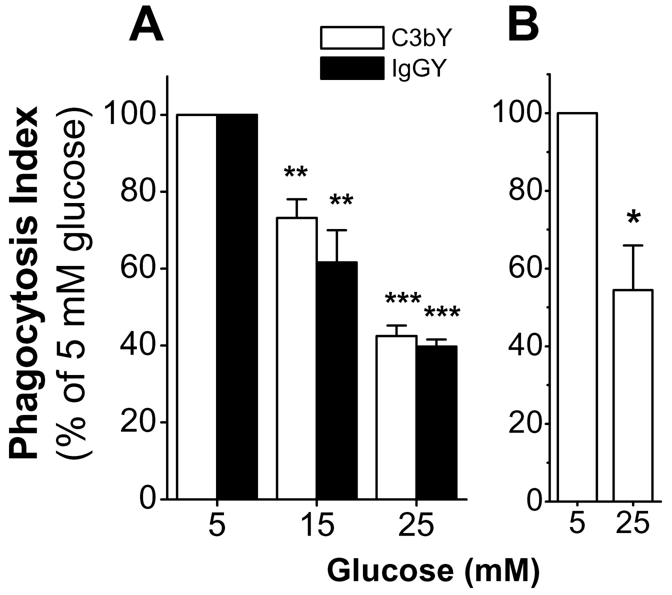

**FIGURE 1**
Elevated D-glucose concentrations inhibit complement receptor– and Fc$\gamma$ receptor–mediated phagocytosis in nonadherent normal human neutrophils. Neutrophils were incubated in suspension with 5, 15, or 25 mM D-glucose in Krebs-Ringer Hepes medium for 30 minutes at 37°C before addition of C3bi- (C3bY) or IgG-opsonized (IgGY) yeast particles (yeast/neutrophil ratio 100:1) for an additional 15 minutes at 37°C. (*A*) Phagocytosis was quantified by light microscopy, as described in Materials and Methods, and expressed as percent of that at 5 mM D-glucose. $^{**}P < .01$ and $^{***}P < .001$, as compared with that at 5 mM glucose, using Student's *t* test for paired comparisons. Values are mean ± SEM for 6 separate experiments. (*B*) Phagocytosis of C3bY quantified by flow cytometry showed virtually similar results. $^{*}P < .05$, as compared with that at 5 mM glucose, using Student's *t* test for paired comparisons. Values are mean ± SEM for 6 separate experiments.

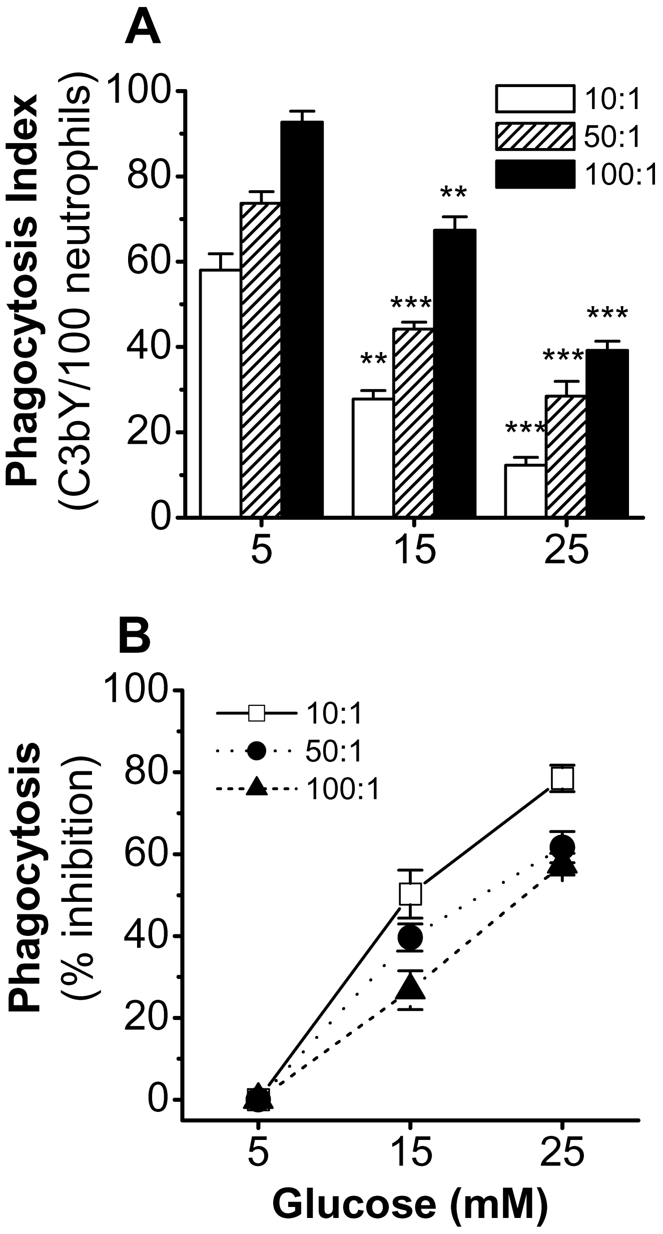

**FIGURE 2**
The yeast/neutrophil ratio does not affect the inhibitory effects of 15 or 25 mM glucose on complement receptor–mediated phagocytosis in nonadherent normal human neutrophils. Neutrophils, preincubated with 5, 15, or 25 mM glucose, were allowed to phagocytose C3bY, as described in the legend to Figure 1, at the indicated yeast/neutrophil ratios. Phagocytosis was quantified by light microscopy and is expressed as (*A*) number of ingested yeast particles/100 neutrophils or (*B*) the relative inhibitory effect of 15 or 25 mM glucose, as compared to that at 5 mM glucose. Quantification of phagocytosis using flow cytometry showed virtually similar results. $^{**}P < .01$ and $^{***}P < .001$, as compared with that at 5 mM glucose, using Student's *t* test for paired comparisons. Values are mean ± SEM for 4 to 6 separate experiments.

**TABLE 1**
Total number of neutrophil-associated yeast particles

| | Glucose (mM) | | |
|---|---|---|---|
| Target | 5 | 15 | 25 |
| C3bY | 265 ± 10 | 248 ± 5 | 232 ± 8* |
| IgGY | 194 ± 12 | ND | 144 ± 37 |

*Note.* Phagocytosis experiments with nonadherent neutrophils in suspension were performed as described in Materials and Methods. For each glucose concentration, all yeast particles associated with the neutrophils, both ingested and bound to the cell surface, were counted. In each experiment, 300 neutrophils were counted in each group. Values are mean ± SEM of the numbers of yeast particles/100 neutrophils in 3 to 6 separate experiments. $^{*}P < .05$ as compared with 5 mM glucose in the same group, using Student's *t* test for paired comparisons. ND = not determined.

acting specifically on the ingestion process following complement or Fc$\gamma$ receptor engagement by particulate stimuli. We therefore decided to further specifically investigate the underlying mechanisms behind the decreased ingestion of C3bY and IgGY.

### Decreased Phagocytosis of C3bY at Increased Glucose Concentration Is Not An Osmotic Effect

To determine whether the inhibitory effects of high glucose concentrations on phagocytosis in neutrophils was an osmotic effect, we examined the effects of the transported but nonmetabolizable glucose analogue 3-oxy-methyl-D-glucose (3-OMG), and of the nontransported stereoisomer L-glucose, on the phagocytosis of C3bY. The results showed no significant changes in phagocytosis by these 2 sugars at 15 or 25 mM, indicating that the effects of high glucose concentrations were not secondary to an osmotic effect (Figure 3).

### Dose-Dependent Inhibitory Effects of PMA on Neutrophil Phagocytosis of C3bY and IgGY

In other cell types, exposure to elevated glucose concentrations may induce cellular dysfunction by the activation of PKC [17, 18]. As the exact role of PKC activation in neutrophil phagocytosis is not fully understood, we next tested the effect of the PKC activator PMA on neutrophil phagocytosis of C3bY and IgGY in our system. The rate of phagocytosis decreased dose-dependently in a similar way for both C3bY and IgGY at 1 nM to 1 $\mu$M PMA (Figure 4). Thus, PKC activation by PMA can decrease the rate of complement or Fc$\gamma$ receptor–mediated phagocytosis of yeast particles in normal human neutrophils.

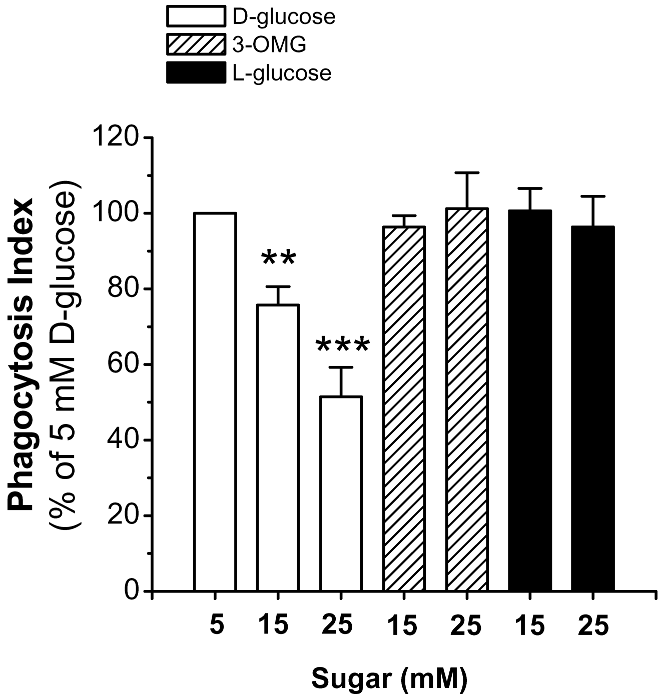

**FIGURE 3**

The inhibitory effect of 15 or 25 mM glucose on neutrophil phagocytosis is not an osmotic effect. Neutrophils were incubated in suspension with increasing concentrations of D-glucose (*open bars*), 3-OMG (*hatched bars*), or L-glucose (*closed bars*) for 30 minutes at 37°C before addition of C3bY (yeast/neutrophil ratio 100:1). In the samples containing 3-OMG and L-glucose, 5 mM D-glucose was mixed with 10 mM or 20 mM 3-OMG and L-glucose as a source of energy. Phagocytosis was scored as described in the legend to Figure 1, and expressed in percent of that at 5 mM D-glucose (56.3 ± 3.3 C3bY/100 neutrophils). $^{**}P < .01$ and $^{***}P < .001$, as compared with that at 5 mM glucose, using Student's $t$ test for paired comparisons. Values are mean ± SEM for 6 separate experiments.

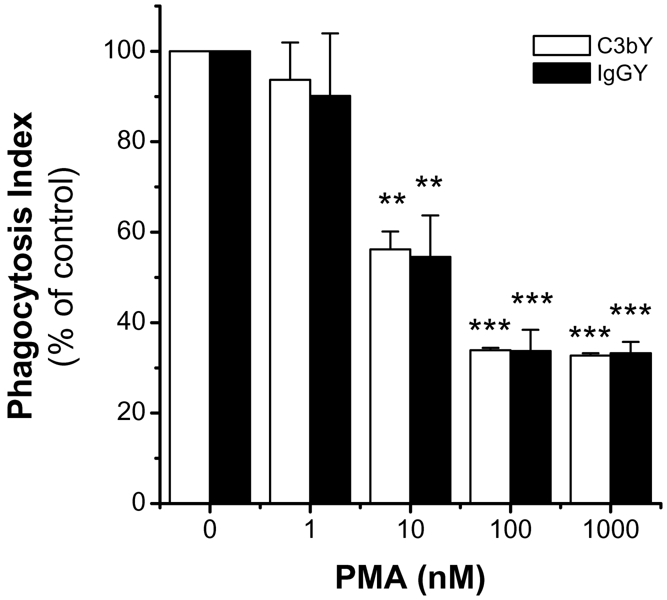

**FIGURE 4**

The PKC agonist PMA can dose-dependently inhibit complement receptor– and Fc$\gamma$ receptor–mediated phagocytosis in normal human neutrophils. Neutrophils were preincubated with 0 to 1000 nM PMA for 5 minutes in suspension, followed by phagocytosis of C3bY or IgGY (yeast/neutrophil ratio 100:1), as described in the legend to Figure 1. Phagocytosis is expressed as percent of that at 5 mM D-glucose. Quantification of phagocytosis using flow cytometry showed virtually similar results. $^{**}P < .01$ and $^{***}P < .001$, as compared with that at 5 mM glucose, using Student's $t$ test for paired comparisons. Values are mean ± SEM for 6 separate experiments.

### The PKC Inhibitors GF109203X or Go6976 Can Reverse the Inhibitory Effects of Elevated Glucose Concentrations on Phagocytosis of C3bY and IgGY

If the glucose-mediated inhibitory effects on phagocytosis of C3bY and IgGY in neutrophils are mediated by PKC activation, a PKC inhibitor should reverse that effect. To investigate the possible inhibitory effects of glucose-mediated PKC activation in phagocytosis of opsonized yeast particles in our system, we tested the effect of 2 specific PKC inhibitors, GF109203X, an inhibitor of the $\alpha$, $\beta$, $\delta$, and $\varepsilon$ isoforms of PKC, and Go6976, an inhibitor of the $\alpha$ and $\beta$ isoforms of PKC. As seen in Figure 5*A*, addition of GF109203X at 10 nM to 1 $\mu$M dose-dependently reversed the inhibitory effect of 25 mM glucose, with an almost normalized phagocytosis at 1 $\mu$M GF109203X. This is in good agreement with our previous findings where 1 $\mu$M of GF109203X could completely abolish PMA-induced superoxide secretion from human neutrophils [19]. In the same way, Go6976 at 0.1 to 10 $\mu$M was also found to dose-dependently reverse the inhibitory effects of 25 mM glucose on neutrophil phagocytosis of both C3bY (Figure 5*B*) and IgGY (data not shown). Also here, we confirmed that 10 $\mu$M Go6976 could completely inhibit PMA-induced superoxide secretion in neutrophils (data not shown). Thus, inhibition of the PKC isoforms $\alpha$ and $\beta$ is sufficient to prevent the decreased rate of phagocytosis seen at elevated glucose concentrations.

### Diacylglycerol Can Inhibit Phagocytosis of C3bY

It has been suggested that activation of PKC can be mediated by de novo synthesis of DAG from glucose [20]. As the PKC isoforms $\alpha$ and $\beta$ can be activated by DAG, we next studied the

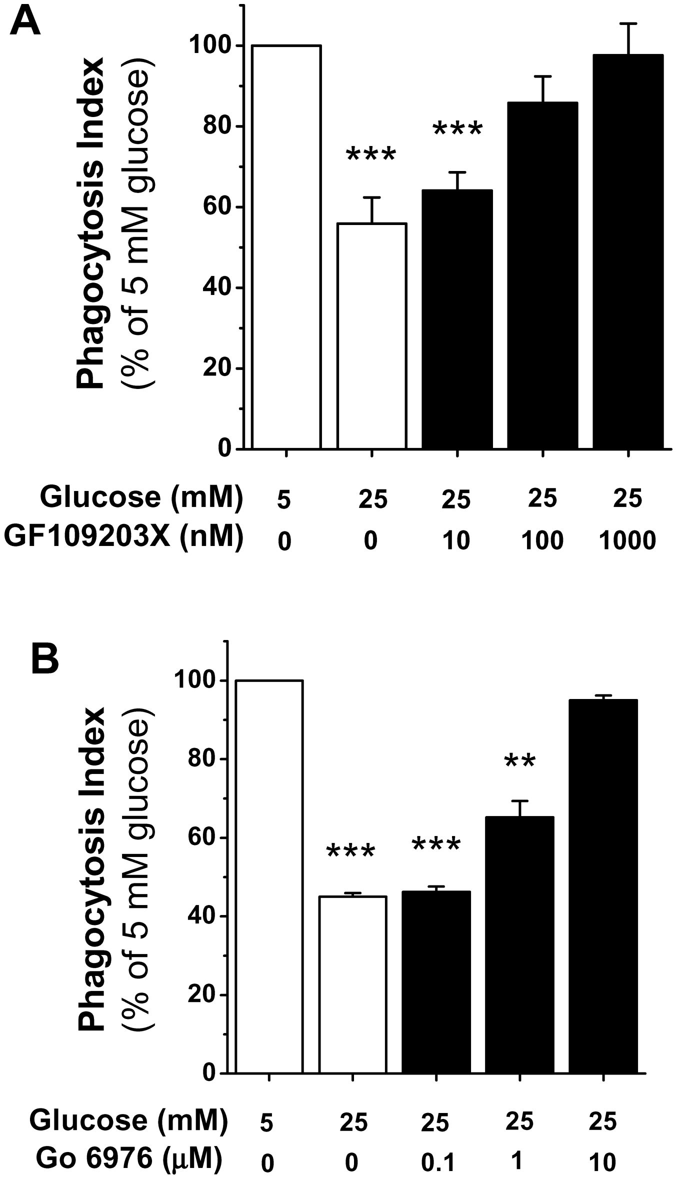

FIGURE 5
The PKC inhibitors GF109203X (*A*) or Go6976 (*B*) can abolish the inhibitory effects of elevated glucose concentrations on neutrophil phagocytosis. Neutrophils were incubated with 5 or 25 mM D-glucose for 30 minutes at 37°C, with or without PKC inhibitors added during the last 5 minutes. C3bY, at a yeast/neutrophil ratio of 100:1, were then added and phagocytosis was allowed to proceed as described in the legend to Figure 1. Phagocytosis is expressed in percent of ingested yeast particles/100 neutrophils in comparison to that at 5 mM D-glucose. Quantification of phagocytosis using flow cytometry showed virtually similar results. $^{**}P < .01$ and $^{***}P < .001$, as compared with that at 5 mM glucose, using Student's *t* test for paired comparisons. Values are mean ± SEM for 6 separate experiments.

capacity of the cell permeable DAG analogue 1,2-dioctanoyl-*sn*-glycerol to inhibit phagocytosis of C3bY. Figure 6*A* shows that DAG dose-dependently inhibited phagocytosis of C3bY, an effect that could be reversed by the PKC inhibitor Go6976. As elevated glucose concentrations and DAG inhibited phagocytosis in a similar manner, we tested for an additive effect by a combination of suboptimal inhibitory concentrations of the 2 substances. Indeed, 10 mM glucose and 0.1 $\mu$M DAG could both inhibit phagocytosis of C3bY by 31.0% ± 5.6% and 31.7% ± 11.6%, respectively, as compaired with that at 5 mM glucose only (Figure 6*B*). The combination of 10 mM glucose and 0.1 $\mu$M DAG further decresed phagocytosis to 57.8% ± 4.4% of that seen at 5 mM glucose only, which was significantly lower than that at 10 mM glucose ($P < .001$) or that at 0.1 $\mu$M DAG, respectively ($P < .05$; Figure 6*B*). Thus, it is possible that the inhibitory effects of elevated glucose concentrations on neutrophil phagocytosis is mediated by DAG-induced activation of PKC$\alpha$ and PKC$\beta$.

### DISCUSSION

The present study demonstrates that an increase of the glucose concentration from 5 mM to 15 or 25 mM can dose-dependently inhibit both complement receptor– and Fc$\gamma$ receptor–mediated phagocytosis in normal human neutrophils. Our results indicate that the glucose-mediated inhibition of phagocytosis is not an osmotic effect of glucose or an impaired ability of the neutrophils to bind yeast particles to the cell surface, but rather a more pronounced effect on the ingestion process.

It is well known that hyperglycemia may lead to PKC activation, which may underly insulin resistance and diabetic complications in different tissues [17, 21]. Several PKC isotypes have been described so far, which fall into different categories based on primary structure and biochemical properties [22, 23]. The PKC isoforms $\beta_1$and $\beta_2$, which require phosphatidylserine (PS) and are activated by $Ca^{2+}$ and DAG, are the most abundant isoforms found in neutrophils [24, 25]. Also other PKC isoforms, such as PKC$\zeta$ and PKC$\delta$, have been found in human neutrophils [22, 24]. However, the exact role of different PKC isoforms in neutrophil function is not known in detail.

Elevation of extracellular glucose can result in an increased PKC activity in the membranous fraction, with a parallel decrease in the cytosolic PKC fraction without alteration of the total PKC activity in endothelial cells [17]. Furthermore, elevation of the blood glucose to more than 11 mM was found to increase the levels of membrane-bound and cytosolic PKC$\beta_2$ in platelets, both from patients with type 2 diabetes mellitus and from healthy controls [18]. We have shown that elevated

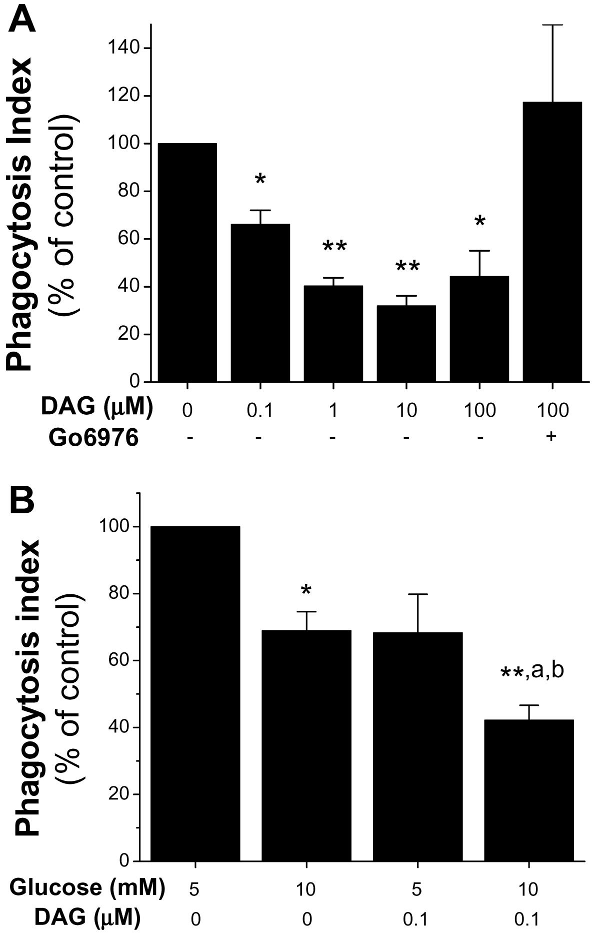

**FIGURE 6**

($A$) The cell permeable DAG analogue 1,2 dioctanoyl-*sn*-glycerol can dose-dependently inhibit complement receptor mediated–phagocytosis in normal human neutrophils. Neutrophils were incubated for 5 minutes at 37°C with 5 mM D-glucose, 0 to 100 $\mu$M DAG and 10 $\mu$M Go6976 as indicated, prior to the addition of C3bY. The neutrophils were allowed to phagocytose C3bY as described in the legend to Figure 1. Phagocytosis is expressed in percent of that at 5 mM glucose only. ($B$) Additive phagocytosis inhibitory effects of suboptimal inhibitory concentrations of glucose and DAG. Neutrophils were incubated for 30 min at 37°C with 5 or 10 mM D-glucose and with 0.1 $\mu$M DAG added during the last 5 minutes prior to the addition of C3bY, as indicated. Phagocytosis was quantified by flow cytometry. Quantification of phagocytosis using light microscopy showed virtually similar results. $^{*}P < .05$ and $^{**}P < .01$, as compared with that in medium with 5 mM glucose and no additives, using Student's $t$ test for paired comparisons. $^{a}P < .05$, as compared with that in samples with 5 mM glucose and 0.1 $\mu$M DAG; $^{b}P < .001$, as compared with that in samples with 10 mM glucose only, using Student's $t$ test for paired comparisons. Values are mean ± SEM for 5 separate experiments.

glucose concentrations can inhibit insulin-induced chemokinesis in normal human neutrophils, an effect that was reversed by the PKC inhibitor GF109203X, suggesting that PKC activation was mediating the inhibitory effect of glucose on insulin-induced chemokinesis [19]. Based on these previous observations, we tested the hypothesis that the inhibitory effect of elevated glucose concentrations on neutrophil phagocytosis in our system was also mediated by glucose-induced PKC activation. This hypothesis was supported by several observations. The PKC activator PMA dose-dependently inhibited neutrophil phagocytosis of both C3bY and IgGY and the specific PKC inhibitors GF109203X (inhibits $\alpha$, $\beta$, $\delta$, and $\varepsilon$ isoforms of PKC) and Go6976 (inhibits the $\alpha$ and $\beta$ isoforms of PKC) each abolished the inhibitory effects of elevated glucose concentrations on phagocytosis of C3bY and IgGY. Thus, glucose-induced PKC activation may also be part of the pathological changes seen in neutrophils in diabetes mellitus.

Previous observations suggested that activation of PKC in diabetes could be mediated by de novo synthesis of DAG from glucose. Such de novo synthesis of DAG has been observed both in endothelial cells [17] and in neutrophils activated by $\beta$-glucan particles [20]. Therefore, it is interesting to note that a cell-permeable DAG analogue also dose-dependently inhibited phagocytosis of C3bY by normal neutrophils, an effect that, like in the case of D-glucose, was completely reversed by the PKC inhibitor Go6976, suggesting that the effect of DAG also was mediated by PKC$\alpha$ and/or PKC$\beta$. The additive effect of suboptimal inhibitory concentrations of glucose and DAG on phagocytosis further indicates a link between elevated glucose concentrations, DAG-mediated activation of PKC, and an inhibitory effect on neutrophil phagocytosis.

Interestingly, a few studies in neutrophils, macrophages and monocytes have shown that activation of PKC is associated with, and sometimes necessary for some isolated steps in the phagocytosis process [15, 26]. On the other hand, other studies showed that glucose-induced activation of PKC could inhibit the generation of cytokines such as interleukin-1 (IL-1) in macrophages [27]. This demonstrates that other cellular functions in phagocytes can also be inhibited by glucose-induced PKC activation. Moreover, a recent study showed that the PKC inhibitor GF109203X increased the rate of phagocytosis in bovine neutrophils [28]. Most studies on the role of PKC activation in the phagocytosis process looked at signaling events very early in the phagocytosis process. In our study design, we evaluated phagocytosis after a 30-minute preincubation with various glucose concentrations, followed by a subsequent 15-minute incubation of neutrophils with opsonized yeast, which may in part explain the discrepancy with respect to PKC dependence. Thus, the exact role of PKC activation in phagocytosis is still to be completely understood.

We conclude that the inhibitory effect of elevated glucose concentrations on phagocytosis of C3bY and IgGY in normal human neutrophils involve activation of PKC$\alpha$ and/or PKC$\beta$, possibly through generation of DAG from glucose. The specific intracellular signaling mechanisms in the neutrophils that are affected by such glucose-mediated PKC activation are still unclear and need further investigation.

### REFERENCES

[1] Geerlings, S. E. (1999) Immune dysfunction in patients with diabetes mellitus. *FEMS Immunol. Med. Microbiol.,* **26,** 259–265.

[2] Wilson, R. M. (1986) Neutrophil function in diabetes. *Diabet. Med.,* **3,** 509–512.

[3] Delamaire, M. (1997) Impaired leucocyte functions in diabetic patients. *Diabet. Med.,* **14,** 29–34.

[4] Ortmeyer, J., and Mohsenin, V. (1996) Inhibition of phospholipase D and superoxide generation by glucose in diabetic neutrophils. *Life Sci.,* **59,** 255–262.

[5] Wilson, R. M., and Reeves, W. G. (1986) Neutrophil phagocytosis and killing in insulin-dependent diabetes. *Clin. Exp. Immunol.,* **63,** 478–484.

[6] Dziatkowiak, H., Kowalska, M., and Denys, A. (1982) Phagocytic and bactericidal activity of granulocytes in diabetic children. *Diabetes,* **31,** 1041–1043.

[7] Tater, D., Tepaut, B., Bercovici, J. P., and Youinou, P. (1987) Polymorphonuclear cell derangements in type I diabetes. *Horm. Metabol. Res.,* **19,** 642–647.

[8] Edwards, S. W. (1994) *Biochemistry and Physiology of the Neutrophil.* Cambridge, UK, Cambridge University Press.

[9] Marhoffer, W., Stein, M., Schleinkofer, L., and Federlin, K. (1994) Monitoring of polymorphonuclear leukocyte functions in diabetes mellitus—a comparative study of conventional radiometric function tests and low light imaging systems. *J. Biolumin. Chemilumin.,* **9,** 165–170.

[10] Bagdade, J. D., Root, R. K., and Bulger, R. J. (1973) Impaired leukocyte function in patients with poorly controlled diabetes. *Diabetes,* **23,** 9–15.

[11] Van Oss, C. J. (1971) Influence of glucose levels on the in vitro phagocytosis of bacteria by human neutrophils. *Infect. Immun.,* **4,** 54–59.

[12] Van Oss, C. J., and Border, J. R. (1978) Influence of intermittent hyperglycaemic glucose levels on the phagocytosis of microorganisms by human granulocytes in vitro. *Immunol. Comm.,* **7,** 669–676.

[13] Pickering, L. K., Cleary, T. J., and Getz, S. (1982) Effect of glucose concentration and time on polymorphonuclear leukocyte and function. *J. Clin. Lab. Immunol.,* **8,** 31–35.

[14] Böyum, A. (1968) Separation of leukocytes from blood and bone marrow. *Scand. J. Clin. Lab. Invest.,* **97,** 77–89.

[15] Andersson, T., Fällman, M., Lew, D. P., and Stendahl, O. (1988) Does protein kinase C control receptor-mediated phagocytosis in human neutrophils? *FEBS Lett.,* **239,** 371–375.

[16] Giaimis, Y., Lombard, Y., Makaya-Kumba, M., Fonteneau, P., and Poindron, P. (1992) A new and simple method for studying the binding and ingestion step in the phagocytosis of yeasts. *J. Immunol. Methods,* **154,** 185–193.

[17] Lee, T. S. (1989) Activation of protein kinase C by elevation of glucose concentration: Proposal for a mechanism in the development of diabetic vascular complications. *Proc. Natl. Acad. Sci. U.S.A.,* **86,** 5141–5145.

[18] Pirags, V., Assert, R., Haupt, K., Schatz, H., and Pfeiffer, A. (1996) Activation of human platelet protein kinase C-$\beta$2 in vivo in response to acute hyperglycemia. *Exp. Clin. Endocrinol.,* **104,** 431–440.

[19] Oldenborg, P. A., and Sehlin, J. (1999) Hyperglycemia in vitro attenuates insulin-stimulated chemokinesis in normal human neutrophils. Role of protein kinase C activation. *J. Leukoc. Biol.,* **65,** 635–640.

[20] Rossi, F., Grzeskowiak, M., Della Bianca, V., and Sbarbati, A. (1991) De novo synthesis of diacylglycerol from glucose. A new pathway of signal transduction in human neutrophils stimulated during phagocytosis of $\beta$-glucan particles. *J. Biol. Chem.,* **266,** 8034–8038.

[21] Pfeiffer, A. (1996) Diabetes: Many leads to PKC (protein kinase C). *Exp. Clin. Endocrinol.,* **104,** 17–18.

[22] Kent, J. D., Sergeant, S., Burns, D. J., and McPhail, L. C. (1996) Identification and regulation of protein kinase C-$\delta$ in human neutrophils. *J. Immunol.,* **157,** 4641–4647.

[23] Dekker, L. V., Leitges, M., Altschuler, G., Mistry, N., McDermott, A., Roes, J., and Segal, A. W. (2000) Protein kinase C-beta contributes to NADPH oxidase activation in neutrophils. *Biochem. J.,* **347,** 285–289.

[24] Thelen, M., Wirthmueller, U. (1994) Phospholipases and protein kinases during phagocyte activation. *Curr. Opin. Immunol.,* **6,** 106–112.

[25] Pongracz, J., and Lord, J. M. (1998) Superoxide production in human neutrophils: Evidence for signal redundancy of more than one PKC isoenzyme class. *Biochem. Biophys. Res. Commun.,* **247,** 624–629.

[26] Raeder, E. M., Mansfield, P. J., Hinkovska-Galcheva, V., Shayman, J. A., and Boxer, L. A. (1999) Syk activation initiates downstream signaling events during human polymorphonuclear-leukocyte phagocytosis. *J. Immunol.,* **163,** 6785–6793.

[27] Hill, R. J., Know, G., Marshall, C. A., and McDaniel, M. L. (1998) Hyperglycemic levels of glucose inhibit Interleukin 1 release from RAW 264.7 murine macrophages by activation of protein kinase C. *J. Biol. Chem.,* **273,** 3308–3313.

[28] Yamamori, T., Inanami, O., Nagahata, H., Cui, Y., and Kuwabara, M. (2000) Roles of p38 MAPK, PKC and PI3-K in the signaling pathways of NADPH oxidase activation and phagocytosis in bovine polymorphonuclear leukocytes. *FEBS Lett.,* **467,** 253–258.

